# Bismuth Oxide Nanoparticle-Enhanced Poly(methyl methacrylate) Composites for I-131 Radiation Shielding: A Combined Simulation and Experimental Investigation

**DOI:** 10.3390/polym17050590

**Published:** 2025-02-23

**Authors:** Suphalak Khamruang Marshall, Kullapat Boonpeng, Nattawat Buapud, Sasikarn Chimhashat, Jarasrawee Chuaymuang, Poochit Kwandee, Nueafa Songphum

**Affiliations:** Department of Radiology, Faculty of Medicine, Prince of Songkla University, Songkhla 90110, Thailand

**Keywords:** bismuth oxide, nanoparticles, poly(methyl methacrylate), composites, I-131 radiation shielding, Phy-X/PSD, nanocomposites, radiation protection, ionizing radiation, PMMA

## Abstract

This study investigates the development of advanced radiation shielding materials incorporating bismuth oxide (Bi_2_O_3_) nanoparticles (NPs) into polymethyl methacrylate (PMMA) composites, comparing efficacy against I-131 gamma radiation. The NPs exhibit a 1.53-fold reduction in z-average diameter and a significantly higher surface area than Bi_2_O_3_, ensuring superior dispersion and structural uniformity within the PMMA matrix. These characteristics, validated through SEM, EDX, and XRD analyses, contribute to enhanced gamma radiation attenuation, leveraging the high atomic number and density of Bi_2_O_3_. Mechanical evaluations reveal that increasing Bi_2_O_3_-NPs concentrations enhances ductility but reduces tensile strength, likely due to nanoparticle agglomeration and stress concentration. Radiation shielding performance, assessed using XCOM and Phy-X/PSD simulations, demonstrates a direct correlation between Bi_2_O_3_ content and attenuation efficiency. Notably, composites with 75% Bi_2_O_3_ content exhibit attenuation properties comparable to, or exceeding, those of PbO_2_, achieving superior shielding efficacy at reduced thicknesses across various photon interaction mechanisms. These findings position Bi_2_O_3_ NPs-enhanced PMMA composites as promising lightweight high-performance alternatives to lead-based shields. By addressing toxicity and environmental concerns associated with lead, this work emphasizes the potential of high-Z nanomaterials in advancing radiation protection applications. This study highlights a transformative approach to designing safer and more efficient shielding solutions, contributing to the next generation of radiation protection materials.

## 1. Introduction

Globally, the use of ionizing radiation in healthcare has grown, driven by its diverse applications and advancements in medical technology [1]. Over the past few decades, there has been substantial progress in the development of new technologies that have resulted in substantial improvements in patient care. However, thyroid cancer has become increasingly prevalent in recent decades. Iodine-131 (I-131) is a crucial radiopharmaceutical used to diagnose and treat thyroid cancer [2]. It functions as the first theranostic agent due to its dual emission capabilities: approximately 90% beta particles, which target and destroy cancer cells for therapeutic purposes, and about 10% gamma particles, utilized for diagnostic imaging [3]. The radioactive decay of I-131 is primarily achieved through beta emission, with a half-life of 8.03 days. The beta particles emitted have an energy of 606 keV and 89.6% abundance. Additionally, the primary gamma emission of I-131 has an energy of 364 keV comprising 81% of its emissions [4]. These I-131 high-energy particles are able to penetrate tissues to a depth of several millimeters, effectively causing cellular damage and inducing cell death, making I-131 particularly effective for targeted radiotherapy in conditions, such as thyroid cancer [5,6], and it has shown to be effective in treating other cancers [7].

Furthermore, the global number of I-131 procedures for diagnostic and therapeutic purposes is significant, indicating its widespread worldwide use in treating thyroid-related conditions. Estimates suggest that millions of procedures are performed annually worldwide. These include diagnostic scans for thyroid function, structure, and therapeutic uses, particularly for thyroid cancer and hyperthyroidism. In the United States alone, tens of thousands of cases are reported annually, primarily for thyroid cancer treatment and hyperthyroidism management [8,9]. Thyroid cancer is ranked in seventh place in 2022 by Globocan, with 821,214 new incidences (72.6% in Asia) and 47,507 mortalities (ranked 47th) [10]. For diagnostic and therapy purposes, a specified dose of I-131 is administered to a patient via oral solution, capsule, or intravenous injection [11]. Several variables affect radioiodine activity, including gender, therapeutic indication, body mass index, and age.

The rising use of I-131 in diagnostic and therapeutic procedures has led to concerns about higher cumulative radiation exposure for medical personnel. Among nuclear medicine technologists, the primary source of exposure to I-131 is radioactive patients rather than the preparation of radiopharmaceuticals [12]. The maximum beta range of I-131 in air is 165 cm; to protect medical staff, portable lead shields or lead fixed barriers are commonly used [13]. Despite the excellent shielding properties of lead and other heavy metals, these materials exhibit significant limitations. These include being bulky, corrosive, heavy, and toxic. It was reported by Burns et al. that of 172 lead shields, between 56 and 70% of the radiation lead shields were found to have surface lead dust [14]. Additionally, they demonstrate inadequate thermal and mechanical stability under operational conditions. Consequently, there is increasing interest in the development of innovative radiation-shielding materials capable of addressing these deficiencies.

The rising interest in radiation shielding polymer composites with high atomic numbers arises from their capacity to deliver effective radiation protection while overcoming the limitations of traditional shielding materials [15,16]. In particular, wearable lightweight polymer composites for radiation shielding are used by medical staff [17,18]. Recent investigations have shown that high-Z materials can be integrated into polymer matrices to improve radiation shielding. Bel et al. investigated the efficiency of colemanite-reinforced poly(methyl methacrylate) (PMMA) composites for radiation attenuation in situations with combined neutron and gamma-ray exposure. Its mechanical properties and strong neutron and gamma radiation suppression made the amalgamation effective for radiation shielding [19]. In addition, Alsaab and Zeghib developed a lightweight lead-free polymer nanocomposite for medical X-ray and gamma protection, which increased attenuation while maintaining polymer matrix flexibility and processability [20]. Rise et al. demonstrated that bismuth’s elevated atomic number and favorable interaction cross-section with gamma radiation enhance the attenuation efficacy of Bi-PMMA composites for low-energy gamma-ray shielding [15]. These investigations show that PMMA-based composites can effectively block radiation, especially when reinforced with high-Z fillers such as colemanite or Bi_2_O_3_.

Recognizing the critical need to safeguard nuclear medicine personnel from radiation exposure, this research developed radiation shielding panels using PMMA doped with Bi_2_O_3_ nanoparticles (Bi_2_O_3_-NPs). The objective was to evaluate the Bi_2_O_3_-NP-enhanced PMMA composite shielding efficacy in attenuating I-131 radiation. This study uniquely combines powerful computational methods, including XCOM and Phy-X/PSD, to assess the shielding efficacy of PMMA composites augmented with Bi_2_O_3_ and Bi_2_O_3_-NPs against I-131 radiation. Additionally, the novel use of anthropomorphic phantoms was utilized to replicate the human anatomy of nuclear medicine workers, enabling the accurate assessment of dose attenuation at different depths.

## 2. Materials and Methods

### 2.1. Materials

Bismuth nitrate pentahydrate (Bi(NO_3_)_3_∙5H_2_O, 98.0% analytical reagent grade) was acquired from KemAus (Cherrybrook, New South Wales, Australia). Sodium hydroxide (NaOH), sodium sulfate (Na_2_SO_4_), and poly(methyl methacrylate) were sourced from Thermo Fisher Scientific (Waltham, MA, USA). Bismuth oxide (99.5% analytical reagent grade) was obtained from Q RëC™ (QRëc Chemical Co., Ltd., Auckland, New Zealand), and lead (IV) oxide (RPE-grade, analytical) was supplied by CARLO ERBA Reagents (Cornaredo, Milano, Italy). Deionized water for all experiments was prepared using a Direct-Q3 water purification system. All solvents employed in this study were procured from Millipore Sigma (St. Louis, MO, USA) and Thermo Fisher Scientific (Waltham, MA, USA).

### 2.2. Preparation of Poly(methyl methacrylate) (PMMA) Composites with Bi_2_O_3_ Nanoparticles

The fabrication of Bi_2_O_3_ nanoparticles can be achieved using several processes, such as chemical precipitation and ultraviolet (UV) curing procedures. Each process has unique benefits and drawbacks that affect the characteristics and usability of the nanoparticles. Chemical precipitation simplifies and controls particle properties, making it ideal for Bi_2_O_3_-NP synthesis. UV curing is fast and energy-efficient, but material compatibility and penetration depth may restrict it; thus, we chose chemical precipitation [21,22].

**Figure 1:**
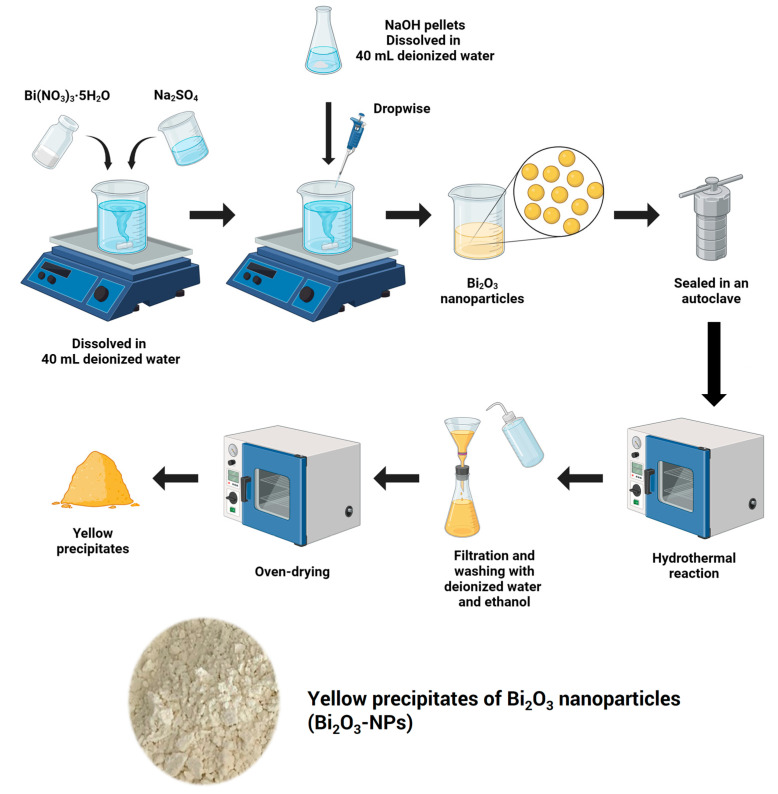
Schematic of the synthesis of Bi_2_O_3_ nanoparticles.

To prepare Bi_2_O_3_-NPs using the chemical precipitation technique, a solution of Bi(NO_3_)_3_∙5H_2_O was first prepared by dissolving it in deionized water to a concentration of 0.2 M. The solution was stirred until the bismuth nitrate was completely dissolved. Next, a 1 M NaOH solution was prepared in deionized water to act as the precipitating agent. The NaOH solution was slowly added to the bismuth nitrate solution while stirring continuously, maintaining the pH of the solution between 7 and 10. As NaOH was added, a white precipitate of bismuth hydroxide (Bi(OH)_3_) formed. The solution was stirred for 2 h to ensure complete precipitation of the bismuth hydroxide. Upon completion of the precipitation process, the solution was filtered using vacuum filtration to collect the precipitate. The precipitate was washed three times with deionized water to remove excess sodium nitrate and other soluble impurities, and it was then washed with ethanol to eliminate any organic impurities. After washing, the precipitate was transferred to a drying oven and dried at 60 °C overnight. Once thoroughly dried, the precipitate underwent thermal decomposition to form Bi_2_O_3_. After cooling, the Bi_2_O_3_-NPs were stored in a dry, airtight container to protect them from moisture and contaminants that could cause oxidation or degradation (Figure 1).

To prepare PMMA composites with Bi_2_O_3_-NPs, pre-polymerized PMMA resin was dissolved in methyl methacrylate. The solution was stirred thoroughly until the PMMA was completely dissolved, forming a homogeneous solution. Once the PMMA solution was ready, the Bi_2_O_3_-NP dispersion was slowly added. The two components were mixed well using a magnetic stirrer to ensure uniform distribution of the nanoparticles throughout the polymer matrix. To initiate polymerization, the benzoyl peroxide polymerization initiator was added to the mixture at 1% based on weight relative to PMMA. After thorough mixing, the mixture was poured into molds for the desired shape. The composite was then cured by heating at 60 °C for 6 h to allow the polymerization of PMMA. The composite was post-cured by heating it at 100 °C for an additional hour to ensure complete cross-linking and removal of any residual solvent or monomers. After polymerization, the composite was allowed to cool and was removed from the mold.

### 2.3. Structural and Morphological Characterization of Poly(methyl methacrylate) (PMMA) Composites with Bi_2_O_3_ Nanoparticles

#### 2.3.1. Characterization of Bi_2_O_3_ Nanoparticles: Size and Zeta Potential Analysis

The samples (Bi_2_O_3_ or Bi_2_O_3_-NPs) were diluted in deionized water to a concentration of 0.5 mg/mL. After dilution, the samples were homogenized in a bath sonicator for 15 min to ensure uniform dispersion of the Bi_2_O_3_ or Bi_2_O_3_-NPs. Following sonication, the samples were filtered through a 0.2 µm PVDF filter to extract any large aggregates or impurities. After preparation of the samples was complete, 1 mL of each sample was transferred into the zeta cell or cuvette for measurement. The temperature control was set to 25 °C prior to initiating the measurement. The dynamic light scattering instrument (ZetaPALS, Brookhaven Instruments Corporation, Nashua, NH, USA) was then configured for size and zeta potential measurements. For size analysis, a scattering angle of 173° was selected, with a refractive index of 1.330 for the fluid and a viscosity of 0.890 cP.

#### 2.3.2. Characterization of Materials Using Scanning Electron Microscopy (SEM) and Energy-Dispersive X-Ray (EDX): Morphology and Composition

Using SEM (FE-SEM Apreo, FEI Technologies Inc., Hillsboro, OR, USA) and EDX (Oxford Instruments NanoAnalysis, High Wycombe, UK), the PMMA, Bi_2_O_3_, and Bi_2_O_3_-NPs were characterized. Therefore, in order to produce excellent image quality and analysis, a meticulous cleaning and preparation process was followed. Firstly, in order to remove any impurities, the PMMA, Bi_2_O_3_, and Bi_2_O_3_-NPs were cleaned with ethanol and then further cleaned for 10–15 min by sonication. On completion of cleaning, the samples were then washed with deionized water prior. To remove any moisture after cleaning, the samples were then placed in a vacuum desiccator. The moisture needs to be removed as the images can be distorted, as any moisture present can lead to charging properties [23]. To overcome this, all the samples were sputter-coated with a 20 nm thin layer of gold, a conductive material [24]. On completion of the gold sputter-coating, all samples were then placed in a vacuum desiccator for 15 min to remove any residual moisture prior to SEM.

Next, the samples were then placed into the SEM chamber for imaging, and the SEM produced high-resolution pictures displaying the surface structure of the samples. To determine the samples’ chemical composition, energy-dispersive X-ray (EDX) analysis was employed, with a particular focus on detecting bismuth and oxygen in Bi_2_O_3_ and the Bi_2_O_3_-NPs, and PMMA matrix composition was analyzed.

#### 2.3.3. X-Ray Diffraction (XRD): Investigating Crystalline and Composite Material Structures

To prepare solid samples of PMMA, Bi_2_O_3_, and Bi_2_O_3_-NPs for XRD analysis, the materials were meticulously ground in an agate mortar and pestle to achieve a fine powder with particle sizes below 10 µm, minimizing any preferred orientation effects. The resulting mixture was then pressed into a uniform, flat pellet using a hydraulic press, applying a pressure of 5 tons for 2 min. The pellet was required to have a smooth, even surface to optimize diffraction quality. After preparation, the samples were loaded into the XRD sample holder. XRD patterns were attained utilizing Cu-Kα radiation (λ = 1.5406 Å) over a scan range of 10–90° 2θ. In order to verify the samples crystalline phases, their peak positions and intensities were assessed against standard databases. The calculation of the Bi_2_O_3_-NPs’ crystallite size was evaluated by applying the Scherrer equation [25]. Additionally, for the bismuth oxide compound, this study used reference code 01-071-0465, with the primary reference and structure derived from the Harwig study [26].

#### 2.3.4. Fourier Transform Infrared Spectroscopy (FTIR) Analysis: Investigating Molecular Structures and Chemical Bonding

To examine the samples’ molecular structures and chemical bonding, Fourier transform infrared spectroscopy (FTIR) analysis was carried out (VERTEX 70, Bruker Daltonics GmbH & Co. KG, Bremen, Germany). We then applied the attenuated total reflection (ATR) technique [27], with the spectra ranging from 4000 cm^−1^ to 400 cm^−1^, with 64 scans per measurement. Additionally, to focus on identifying specific chemical bonds, all of the measurements were taken using a deuterated l-alanine-doped triglycine sulfate (DLATGS) detector (Leonardo Electronics Us Inc., McLean, VA, USA) with potassium bromide (KBr) precision windows. Moreover, the FTIR analysis provided a valuable understanding of the composite samples. To detect changes in the samples’ chemical structure, the appearance or disappearance of particular bonds was tested.

#### 2.3.5. Mechanical Property Analysis of Composite Materials Using Tensile Testing

Tensile tests were conducted on PbO_2_, PMMA, Bi_2_O_3_, and Bi_2_O_3_ nanoparticle specimens in accordance with the ISO 37 Type II standard [28]. The original dumbbell-shaped test pieces were trimmed to the specified dimensions prior to testing. The tests were conducted with a preload of 0.1 N, a tensile loading of 1 kN, at a test speed of 100 mm/minutes, and a gauge length with a standard travel of 20 mm was used to characterize the mechanical properties of the materials. A universal testing machine (ZwickRoell Z010, Zwick GmbH & Co. KG, Ulm, Germany) was used for the experiments, and the ultimate tensile strength and elongation break were determined.

### 2.4. Radiation Shielding Simulation and Measurements

#### 2.4.1. XCOM-Based Simulation of Materials for Radiation Shielding

The shielding characteristics, including coherent scattering, incoherent scattering, photoelectric absorption, pair production in nuclear field, pair production in electron field, total attenuation with coherent scattering, and total attenuation without coherent scattering, were ascertained utilizing the XCOM algorithm, and the results were compared with EPICS2017 values [29,30]. XCOM generates total cross-sections, attenuation coefficients, and partial cross-sections on a conventional energy grid (spaced logarithmically), a user-selected grid, or a mix of both grids. Of note, a Windows version of XCOM affords a user interface that expedites identifying and defining substances [31].

#### 2.4.2. Phy-X/PSD-Based Simulation of Materials for Radiation Shielding

In this study, the Phy-X/PSD program was applied to calculate the attenuation coefficient [32]. The mass attenuation coefficient (MAC) is a fundamental parameter used to characterize a material’s ability to attenuate I-131 gamma radiation per unit mass. It offers a density-independent measure of shielding effectiveness and is calculated using Equation (1), where the linear attenuation coefficient is presented as μ and ρ is the material’s density. A higher mass attenuation coefficient corresponds to a thinner material required to achieve significant radiation attenuation.

(1) $$MAC=\frac{\mu }{\rho }$$

The linear attenuation coefficient (LAC) (μ) quantifies a material’s ability to attenuate gamma radiation per unit thickness, determined by its atomic composition, density, and the incident photons energy. It describes the exponential attenuation of radiation, calculated by Beer–Lambert’s law [33], as expressed in Equation (2), where LAC is the linear attenuation coefficient, $I_{0}$ is the initial radiation intensity, I is the transmitted intensity, and t the material’s thickness. A higher LAC corresponds to a reduced material thickness required to achieve significant radiation attenuation.

(2) $$LAC(\mu )=\frac{1}{t}ln\left(\frac{I_{0}}{I}\right)$$

The half-value layer (HVL) is a metric indicating the thickness of a material required to diminish radiation intensity by fifty percent, and calculated using Equation (3) [34], where μ (cm^−1^) is the LAC.

(3) $$HVL=\frac{\ln\left(2\right)}{\mu }$$

The tenth value layer (TVL) is the shield thickness to attenuate a radiation beam to 10% of its radiation level and is expressed in Equation (4), where μ is the LAC of the material.

(4) $$TVL=\frac{ln(10)}{\mu }$$

The mean free path (MFP) represents the average distance traveled by a particle—such as a molecule, atom, or photon—before undergoing a collision with another particle. It is inversely proportional to the material’s density and cross-sectional area and can be determined using Equation (5).

(5) $$MFP=\frac{1}{\mu }$$

The effective electron density (N_eff_) refers to the average number of electrons per unit volume within a material, which directly influences its interaction with ionizing radiation. This parameter is fundamental in characterizing a material’s ability to attenuate radiation, as it determines the probability of photon interactions such as Compton scattering, pair production, and photoelectric effect, and calculated using Equation (6), where Z is the material atomic number, A the material atomic weight, ρ is the mass density, $N_{A}$ is Avogadro’s number (6.022 × 10^23^ atoms/mol), $w_{i}$ is the weight fraction of the i-th element, and $Z_{i}$ is the atomic number of the i-th element.

(6) $$N_{eff}=\frac{N_{A}\rho }{A}\sum_{i}w_{i}Z_{i}$$

Effective conductivity (C_eff_) characterizes the electrical conductivity of a material by integrating its intrinsic properties with the contributions of charge carriers, as defined by the Drude model [35]. It serves as a measure of the material’s capacity to conduct electric current, influenced by critical factors such as electron density, elementary charge, relaxation time, and electron mass, and is calculated using Equation (7), where C_eff_ is the effective electrical conductivity, N_eff_ is the effective number density of charge carriers, ρ is the material density, e is the elementary charge of an electron (1.602 × 10^−19^ C), τ is the relaxation time (average time between collisions of electrons), and $m_{e}$ is the mass of an electron (9.109 × 10^−31^ kg).

(7) $$C_{eff}=\frac{N_{eff}\rho e^{2}\tau }{m_{e}}\times 10^{3}$$

The atomic cross-section (ACS) defines the probability of interaction between a photon and an atom, such as absorption, ionization, or scattering, determined by the atomic structure and the photon’s energy. The cross-section measures an atom’s effective “target area” for a photon interaction, and is calculated using Equation (8) [36], where σ(E) is the atomic cross-section, E is the energy of the photon, and Photon flux is the number of photons passing through a unit area per second.

(8) $$\sigma \left(E\right)=\frac{Number\;of\;interactions\;per\;unit\;time}{Photon\;flux}$$

Electronic cross-section (ECS) is the photon energy electronic cross-section that measures the likelihood of interactions between a photon and an electron. These interactions can involve scattering, whether elastic or inelastic, or absorption. The ECS is subject to the photon energy and specific interaction mechanisms and calculated using Equation (9), where ${\left(\frac{\mu }{\rho }\right)}_{i}$ is the MAC of the i-th element in the molecule, $w_{i}$ is the weight fraction of the i-th element in the molecule, and $Z_{i}$ is the atomic number of the i-th element.

(9) $$ECS=\frac{\sum_{i}w_{i}\frac{{\left(\frac{\mu }{\rho }\right)}_{i}}{Z_{i}}}{\sum_{i}\frac{w_{i}}{Z_{i}}}$$

The effective atomic number (Z_eff_) is used to represent the composite behavior of a compound or mixture as if it were a single-element material. It is the constituent elements’ atomic numbers’ weighted average and accounts for their relative proportions and interaction mechanisms. The Z_eff_ is particularly relevant in contexts such as radiation shielding and is calculated using Equation (10), where $Z_{i}$ is the atomic number of the i-th element, $w_{i}$ is the weight fraction of the i-th element, and n is the empirical exponent, which depends on the interaction mechanism and photon energy.

(10) $$Z_{eff}=\left(\frac{\sum_{i}w_{i}Z_{i}^{n}}{\sum_{i}w_{i}}\right)\frac{1}{n}$$

The equivalent atomic number (Z_eq_) represents the photon interaction characteristics of a material or mixture, particularly when it mimics the behavior of a single element under specific conditions. Unlike the Z_eff_, which is more general, Z_eq_ is highly application-specific and depends on the photon interaction mechanism, the material composition, and the energy range of interest, and is calculated using Equation (11), where $\mu_{compound}\left(E\right)$is the LAC of the compound or mixture at photon energy E and $\mu_{element}(E,Z_{eq})$is the LAC of a pure element with atomic number $Z_{eq}$ at the same photon energy E.

(11) $$\mu_{compound}\left(E\right)=\mu_{element}(E,Z_{eq})$$

#### 2.4.3. Experimental Analysis of Materials for I-131 Radiation Shielding

**Figure 2:**
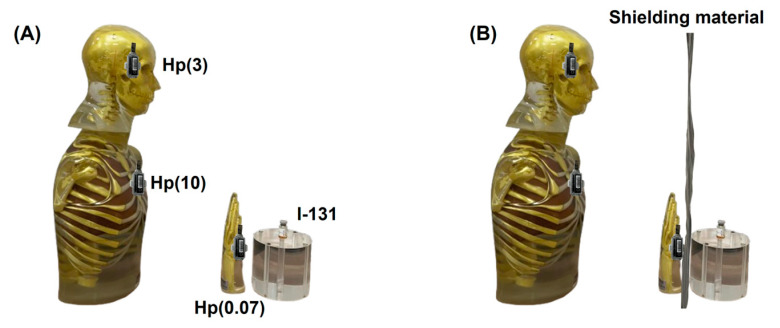
Experimental setup for evaluating materials used in I-131 radiation shielding. The configuration incorporates an RSD anthropomorphic phantom to simulate human tissue interactions, with shielding materials strategically positioned to assess attenuation properties. Radiation dosimeters (Hp(3), Hp(10), and Hp(0.07)) are mounted at critical locations on the phantom to measure dose equivalents, providing insights into the effectiveness of the shielding material under various conditions. (**A**) Setup without shielding material. (**B**) Setup with shielding material.

The equipment utilized in this study comprised Quixel optically stimulated luminescence (OSL) badges (Landauer Inc., Glenwood, IL, USA) in conjunction with I-131 radiopharmaceuticals supplied by the Thailand Institute of Nuclear Technology (Public Organization), Ongkarak, Nakornnayok, Thailand. Anthropomorphic phantoms were employed to simulate the human anatomy of nuclear medicine personnel. Specifically, an RSD anthropomorphic head phantom, including a complete cervical spine (C1–C7), was used for Hp(3) eye lens dosimetry at a tissue depth of 3 mm. An RSD anthropomorphic thorax phantom was utilized to evaluate Hp(10), the whole-body personal dose equivalent, at a depth of 10 mm. Additionally, an anthropomorphic hand phantom (Universal Medical Inc., Oldsmar, FL, USA) was employed for shallow-dose measurements (Hp(0.07)) corresponding to the skin, hands, and feet at a depth of 0.07 mm. These phantoms enabled the assessment of the dose response of OSL dosimeters to I-131 exposure (Figure 2).

For gamma radiation shielding evaluation specific to I-131, Inlight OSL (Quixel) badges were deployed. Effective dose measurements were conducted for Hp(10), Hp(3), and Hp(0.07), addressing the whole body, eye lens, and shallow tissues, respectively. To ensure calibration consistency within the energy range typical of nuclear medicine applications, a traceable ^137^Cs source (662 keV) was used [37]. The calibration protocol adhered to the standards outlined by the Japanese OSL Dosimeters Standards Association [38], including uncertainty determination methods [39]. The Quixel OSL badge dosimeters provided personal dose measurements for Hp(10), Hp(3), and Hp(0.07) in increments of 0.01 mSv, with doses below 0.005 mSv recorded as <0.01 mSv.

### 2.5. Statistical Analysis

In this investigation, each experiment was independently repeated three to ten times, with the results expressed as mean ± standard deviation. The normality of the data was assessed by analyzing residuals, and the homogeneity of variances across groups was verified. Statistical significance was evaluated using Student’s *t*-test, along with one-way or two-way analysis of variance (ANOVA), followed by the Student–Newman–Keuls post hoc test. Significance thresholds were set as follows: ns for *p* > 0.05 and * for *p* ≤ 0.05. All statistical analyses were conducted using GraphPad Prism version 10.0 (GraphPad Software Inc., Boston, MA, USA).

## 3. Results

### 3.1. Structural and Morphological Characterization of Bi_2_O_3_ Nanoparticle-Enhanced PMMA

#### 3.1.1. Bi_2_O_3_ Nanoparticles Characterization: Size and Zeta Potential

**Figure 3:**
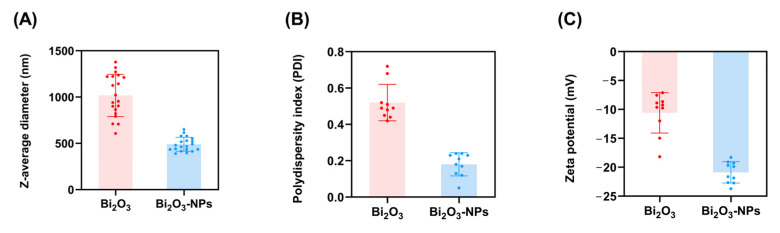
Physicochemical characterization of bismuth oxide (Bi_2_O_3_) and bismuth oxide nanoparticles (Bi_2_O_3_-NPs). (**A**) Z-average diameter (nm); (**B**) polydispersity index (PDI); (**C**) zeta potential (mV). The data are represented as mean ± standard deviation, with *n* = 20, and individual data points plotted.

This study investigates the potential of Bi_2_O_3_-NPs to enhance PMMA composites for radiation shielding materials, specifically for I-131. The findings from Figure 3 indicate that Bi_2_O_3_-NPs exhibit a 1.53-fold smaller z-average diameter compared to Bi_2_O_3_, resulting in a markedly increased surface area. Furthermore, the polydispersity index (PDI) values further confirm that Bi_2_O_3_-NPs demonstrate a narrower particle size distribution relative to Bi_2_O_3_. This property ensures greater uniformity within the PMMA matrix, reducing the likelihood of structural inconsistencies or defects that could compromise the material’s radiation attenuation efficiency. Additionally, the zeta potential analysis indicates that Bi_2_O_3_-NPs possess a significantly more negative zeta potential than their bulk counterparts, indicating superior colloidal stability [40]. Negative zeta potentials provide significant electrostatic repulsion forces among particles, inhibiting aggregation and maintaining solvent dispersion. As a result, this enhanced colloidal stability minimizes nanoparticle aggregation, ensuring consistent and effective distribution throughout the polymer matrix, which is essential for maximizing the material’s shielding performance.

#### 3.1.2. Bi_2_O_3_ Nanoparticle-Enhanced PMMA Composites: SEM and EDX Findings on Morphology and Composition

**Figure 4:**
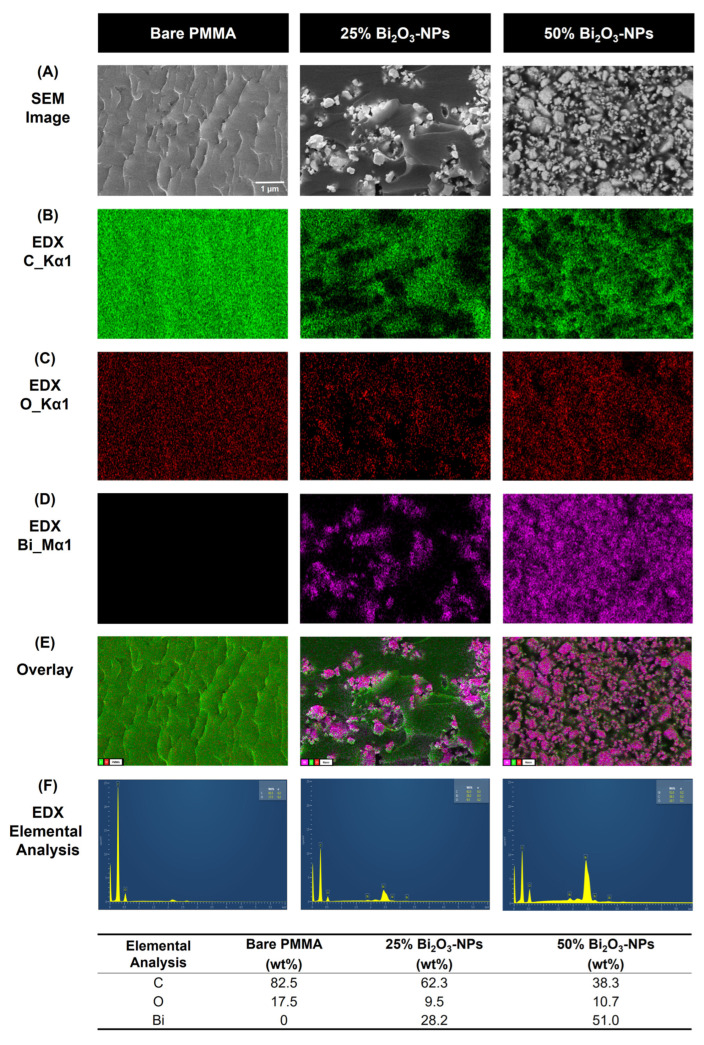
Morphological and elemental analyzes of bare PMMA and Bi_2_O_3_ NP-enhanced PMMA composites were conducted using scanning electron microscopy (SEM) and energy-dispersive X-ray spectroscopy (EDX). (**A**) SEM images of bare PMMA, 25% Bi_2_O_3_ NP-enhanced PMMA, and 50% Bi_2_O_3_ NP-enhanced PMMA, with magnification at 5000× and a scale bar of 1 μm. (**B**–**D**) The EDX elemental maps for carbon (C), oxygen (O), and bismuth (Bi), respectively. (**E**) The overlay maps illustrate the spatial distribution of the elements. (**F**) The EDX spectra, which provide quantitative elemental analysis. The table summarizes the elemental composition (wt%) of C, O, and Bi in bare PMMA and Bi_2_O_3_-NPs.

The morphological and elemental analyses of bare PMMA and Bi_2_O_3_ NP-enhanced PMMA composites demonstrate the successful incorporation of Bi_2_O_3_-NPs into the polymer matrix, as presented by SEM and EDX characterization (Figure 4). The SEM images show that bare PMMA exhibits a smooth and homogeneous surface, whereas the addition of Bi_2_O_3_-NPs significantly alters the surface morphology. Moreover, the SEM images of the composites containing 25% and 50% Bi_2_O_3_-NPs indicate a progressive increase in surface roughness and nanoparticle agglomeration, particularly at higher nanoparticle loadings. These structural changes indicate the successful integration of Bi_2_O_3_-NPs into the PMMA matrix.

Additionally, the EDX elemental maps confirm the presence and distribution of carbon (C), oxygen (O), and bismuth (Bi) in the composites. The maps display a uniform distribution of Bi in the 25% Bi_2_O_3_-NP-enhanced PMMA composite. In comparison, regions of higher Bi concentration are evident in the 50% composite and consistent with the nanoparticle agglomeration observed in SEM images. Furthermore, the overlay images illustrate the spatial distribution of the elements, with increasing Bi signal intensity correlating with higher nanoparticle loading.

The results of the quantitative EDX elemental analysis illustrate significant changes in the composition of the composites. While bare PMMA primarily consists of carbon (82.5 wt%) and oxygen (17.5 wt%), the incorporation of Bi_2_O_3_-NPs introduces bismuth, with its content increasing from 28.2 wt% in the 25% composite to 51.0 wt% in the 50% composite. This increase in Bi content corresponds directly to the expected enhancement in gamma radiation shielding properties, given bismuth’s high atomic number and strong attenuation capabilities [41]. In addition, these results demonstrate that the integration of Bi_2_O_3_-NPs effectively modifies the morphology and elemental composition of PMMA, highlighting its potential as a promising composite material for I-131 radiation shielding applications.

#### 3.1.3. XRD Characterization: Structural Insights into Bi_2_O_3_ Nanoparticle-Enhanced PMMA

**Figure 5:**
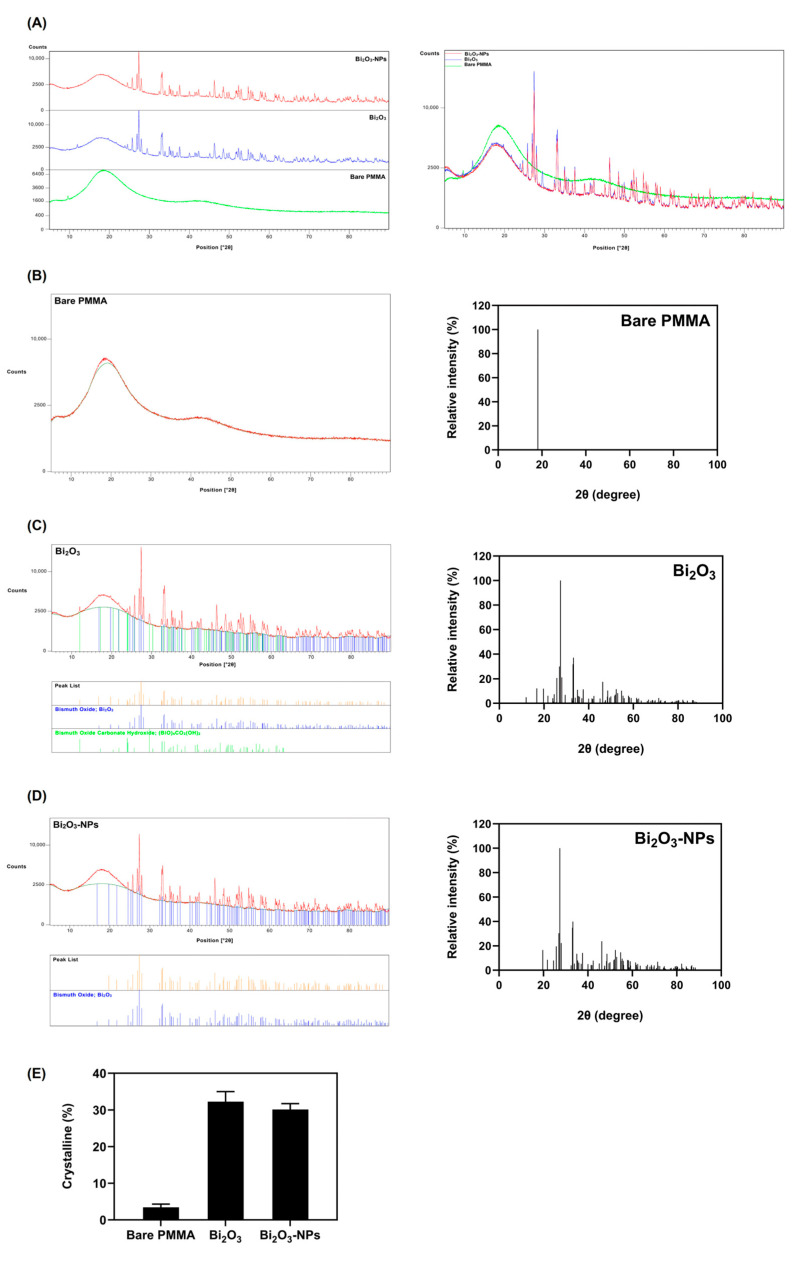
X-Ray diffraction (XRD) analysis and crystallinity evaluation of bare PMMA, Bi_2_O_3_, and Bi_2_O_3_ nanoparticles (Bi_2_O_3_-NPs). (**A**) XRD overlay patterns of bare PMMA, Bi_2_O_3_, and Bi_2_O_3_-NPs. (**B–D**) XRD patterns and relative intensity (%) of diffraction patterns for bare PMMA, Bi_2_O_3_, and Bi_2_O_3_-NPs, respectively. (**E**) Crystallinity percentage comparison of bare PMMA, Bi_2_O_3_, and Bi_2_O_3_-NPs. Data represented as mean ± standard deviation (*n* = 3).

The XRD analysis highlights the structural differences between bare PMMA, Bi_2_O_3_, and Bi_2_O_3_-NPs (Figure 5). In contrast to crystalline materials, polymers like PMMA have amorphous diffraction patterns owing to uneven molecular packing [42]. The XRD pattern for bare PMMA indicates a broad diffraction peak in the 2θ range of 15–25°, characteristic of its amorphous nature, and supports previous investigation on its structural features [20,43]. The non-crystalline characteristics of bare PMMA shows a lack of long-range chains, resulting in the broad peak. This absence of sharp peaks confirms the lack of crystalline domains, which is consistent with the non-crystalline structure of bare PMMA. This amorphous structure is crucial for its flexibility and ease of processing.

In contrast, the XRD pattern of Bi_2_O_3_ exhibits sharp and well-defined peaks, indicative of a high degree of crystallinity. The α-phase of Bi_2_O_3_ contains well-ordered bismuth and oxygen atoms, resulting in discrete lattice planes that diffract X-rays at precise angles and strong XRD peaks. The crystal lattice’s symmetry and periodicity reduce structural flaws, sharpening the peak. The peaks align with standard diffraction patterns of crystalline Bi_2_O_3_, confirming the material’s structural integrity and ordered crystal lattice. Furthermore, the inherent crystal structure and careful control of extrinsic synthesis conditions produce the crisp, well-defined peaks seen in its XRD patterns. A high degree of crystallinity is mostly dependent on phase purity, optimal synthesis conditions, and control of crystallite size, as this XRD study reflects. This high crystallinity enhances Bi_2_O_3_’s potential for functional applications, particularly in radiation shielding due to well-ordered atomic arrangement, enhancing density, structural stability, and interaction with ionizing radiation. Gamma radiation attenuation depends on interactions with dense atomic structures, such as Compton scattering and photoelectric effects. Consequently, the increased density markedly enhances its shielding efficacy.

Furthermore, the Bi_2_O_3_-NPs retain the characteristic crystalline peaks of Bi_2_O_3_ but show noticeable peak broadening and reduced intensity (Figure 5D). This broadening is attributed to the nanoscale size of the particles, which introduces lattice strain and reduces the crystallite size. These nanoscale effects validate the successful synthesis of Bi_2_O_3_ in nanoparticulate form, a key factor in enhancing its functional properties. In addition, the crystallinity analysis further supports these findings. Bare PMMA demonstrates a low crystallinity percentage due to its inherently amorphous nature. However, Bi_2_O_3_ exhibits the highest crystallinity, while Bi_2_O_3_-NPs show a slightly reduced crystallinity. The slight decrease in crystallinity of the Bi_2_O_3_-NPs is because of their increased surface area and energy, smaller particle size, and structural flaws introduced during synthesis. These forces break the long-range order of the crystal lattice, differentiating the structural features of nanoparticles from those of bare PMMA. Additionally, defects in the crystal lattice, including vacancies, interstitials, and dislocations can be introduced into the crystal lattice by fast nucleation and growth processes in the synthesis of nanoparticles. These defects can change the ordered arrangement of atoms, reducing crystallinity. Studies on nanoparticle nucleation and growth processes have shown that rapid nucleation rates might result in structure defects because of kinetic elements surpassing thermodynamic stability [44]. In addition, research shows that the thermally triggered surface nucleation of dislocations fails for 10–50 nm nanoparticles. Geometric effects like increased surface curvature decreases the barrier for dislocation nucleation, and surface diffusion occurs faster on smaller particles, accelerating the formation of surface kinks that nucleate dislocations [45].

#### 3.1.4. FTIR-Based Chemical Bonding Analysis of Bi_2_O_3_ Nanoparticle-Enhanced PMMA Composites

**Figure 6:**
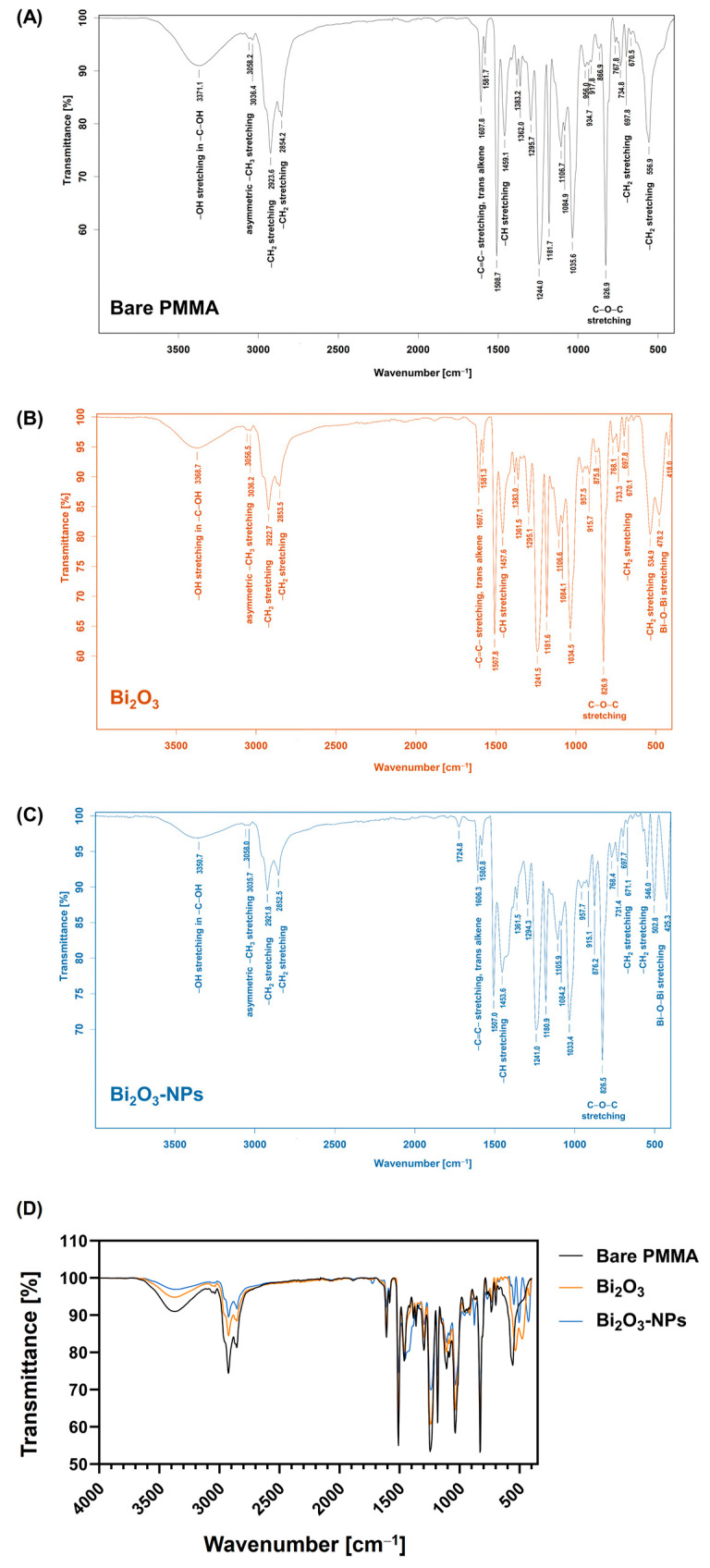
Fourier transform infrared (FTIR) spectra were recorded using the attenuated total reflection (ATR) technique for (**A**) bare PMMA, (**B**) Bi_2_O_3_, (**C**) Bi_2_O_3_ nanoparticles (Bi_2_O_3_-NPs), and (**D**) an overlay of bare PMMA, Bi_2_O_3_, and Bi_2_O_3_ nanoparticles (Bi_2_O_3_-NPs).

The FTIR spectrum of bare PMMA reveals characteristic absorption bands corresponding to its functional groups, providing insights into its chemical structure. A broad band at 3371.1 cm^−1^ suggests hydroxyl (–OH) groups, likely from moisture or surface modifications. Peaks at 3068.2 cm^−1^ and 3036.4 cm^−1^ indicate asymmetric methyl (–CH_3_) stretching, characteristic of PMMA’s backbone, while bands at 2932.6 cm^−1^ and 2854.2 cm^−1^ confirm methylene (–CH_2_) and symmetric methyl stretching, reflecting its aliphatic nature. A peak at 1607.8 cm^−1^ suggests trans-alkene structures, possibly from impurities or byproducts. The strong carbonyl (C=O) stretching peak at 1724.0 cm^−1^, along with C–O–C stretching vibrations at 1244.0 cm^−1^, 1146.2 cm^−1^, and 1084.9 cm^−1^, highlights PMMA’s ester linkages. Absorptions from 934.7 cm^−1^ to 697.8 cm^−1^ correspond to CH_2_ rocking and bending vibrations, while the 555.9 cm^−1^ band indicates CH_2_ out-of-plane bending, potentially related to crystallinity. Key functional groups, including –CH_3_, –CH_2_, C=O, and C–O–C, align with PMMA’s structure, with prominent ester peaks emphasizing its mechanical properties and transparency. Minor hydroxyl and trans-alkene features may reflect slight degradation or residual synthesis impurities (Figure 6).

Additionally, the FTIR spectrum of Bi_2_O_3_ reveals key absorption bands indicative of its chemical structure and interactions. A broad peak around 3368.7 cm^−1^ suggests hydroxyl (–OH) stretching, pointing to surface-adsorbed water or hydroxyl groups from environmental exposure or synthesis conditions. Peaks at 3056.5 cm^−1^ and 3036.2 cm^−1^ correspond to methyl (–CH_3_) stretching, while 2922.7 cm^−1^ and 2853.5 cm^−1^ indicate methylene (–CH_2_) stretching, from organic residues or stabilizing agents. The 1607.1 cm^−1^ peak is attributed to C=C stretching in trans-alkenes, signaling minor organic contamination or precursor remnants. Absorption bands at 1457.6 cm^−1^ and 1383.0 cm^−1^ indicate CH bending, suggesting aliphatic hydrocarbons. Strong bands at 1241.5 cm^−1^, 1181.6 cm^−1^, and 1084.1 cm^−1^ represent C–O–C stretching, from esters or organic components. Bi–O–Bi stretching confirmed by a band at 534.9 cm^−1^, while the broad absorption around 448.8 cm^−1^ supports Bi–O bonds. Additional features in the 915.7–697.8 cm^−1^ range highlight CH_2_ rocking and bending vibrations, indicating organic residues.

The FTIR spectrum of Bi_2_O_3_-NPs provides insights into the functional groups and chemical interactions in the material. A broad band at 3350.7 cm^−1^ indicates hydroxyl (–OH) stretching, linked to surface-adsorbed water or hydroxyl groups. Peaks at 3038.8 cm^−1^ and 3035.7 cm^−1^ represent methyl (–CH_3_) stretching, while bands at 2921.8 cm^−1^ and 2852.5 cm^−1^ correspond to methylene (–CH_2_) stretching, possibly from organic stabilizers. A sharp peak at 1724.8 cm^−1^ suggests carbonyl (C=O) stretching, likely from residual organic compounds. The band at 1606.3 cm^−1^ (C=C stretching) and peaks at 1453.6 cm^−1^ and 1361.5 cm^−1^ (CH bending) indicate minor organic residues. Absorption bands at 1241.0 cm^−1^, 1180.9 cm^−1^, and 1084.2 cm^−1^ reveal C–O–C stretching, highlighting ester linkages. A peak at 546.0 cm^−1^ confirms Bi–O–Bi stretching, with broader absorption at 452.5 cm^−1^ supporting Bi–O bonds. CH_2_ vibrations between 914.5 cm^−1^ and 671.1 cm^−1^ suggest organic residues or stabilizing agents.

#### 3.1.5. Bi_2_O_3_ Nanoparticle-Enhanced PMMA Composites: Tensile Testing Insights into Mechanical Properties

**Figure 7:**
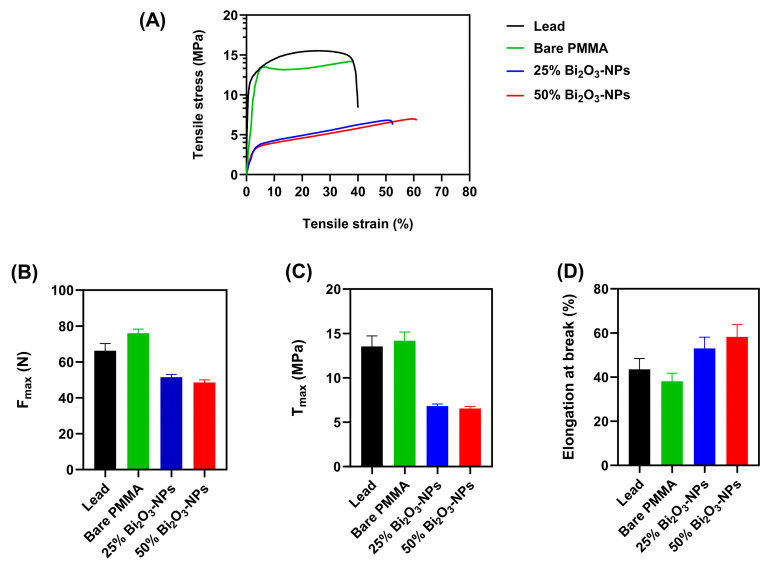
Tensile properties of Bi_2_O_3_ nanoparticle-enhanced PMMA composites. (**A**) Tensile stress–strain curves showing the behavior of lead, bare PMMA (black), 25% Bi_2_O_3_-NPs (blue), and 50% Bi_2_O_3_-NPs (red). (**B**) Maximum force (F_max_) at failure for the different composites. (**C**) Maximum tensile stress at failure (T_max_) for lead, bare PMMA, and Bi_2_O_3_ nanoparticle composites. (**D**) Fracture strain (elongation at break) of the composites. Data represent mean ± standard deviation (*n* = 3).

The mechanical properties of PMMA composites enhanced with Bi_2_O_3_-NPs were evaluated at 25 °C through tensile testing, examining key parameters such as maximum force (F_max_), maximum tensile stress at failure (T_max_), and fracture strain (elongation at break). The data in Figure 7 highlight the effects of varying Bi_2_O_3_ NP concentrations (25% and 50%) on the material’s mechanical behavior. The tensile stress–strain curve (Figure 7A) applying a tensile loading of 1 kN demonstrates distinct differences in the behavior of bare PMMA and the Bi_2_O_3_ NP-enhanced PMMA composites. The bare PMMA exhibits typical brittle fracture behavior, with a sharp increase in tensile stress followed by an abrupt failure. In contrast, the 25% and 50% Bi_2_O_3_-NP-enhanced PMMA composites show a reduction in both tensile stress and strain, suggesting a decrease in material rigidity. Furthermore, the 50% Bi_2_O_3_-NPs composite exhibits a slightly reduced tensile stress and strain compared to the 25% Bi_2_O_3_-NPs composite, indicating a reduction in the material’s ductility and overall load-bearing capacity.

Additionally, Figure 7B illustrates the F_max_ for the various composites. Bare PMMA sustains the highest force of 76.03 N, followed by the 25% Bi_2_O_3_-NPs composite at 51.54 N, and the composite containing 50% Bi_2_O_3_-NPs demonstrates a lower maximum force of 48.96 N. The observed reduction in F_max_ with increasing nanoparticle concentration suggests that incorporating Bi_2_O_3_ may alter the composite’s ability to withstand applied forces, potentially due to the high concentration of nanoparticles within the polymer matrix, which could introduce stress concentrators and reduce overall strength.

Moreover, the maximum tensile stress (T_max_) for bare PMMA is approximately 14.18 MPa (Figure 7C). In contrast, the T_max_ of the 25% Bi_2_O_3_-NPs composite decreases to 6.81 MPa, while the 50% Bi_2_O_3_-NPs composite exhibits a further decrease to 6.54 MPa. This reduction is consistent with the hypothesis that the addition of Bi_2_O_3_-NPs weakens the polymer matrix, likely due to a less effective interaction between the nanoparticles and the polymer chains, leading to reduced stress transfer efficiency and lower tensile strength. Research indicates that adding Bi_2_O_3_ nanoparticles to PMMA might enhance its tensile strength. Mahmood et al. discovered that adding Bi_2_O_3_:Fe_2_O_3_ nanoparticles to PMMA composites at various weight percentages (0.5%, 1%, 3%, and 5%) increased their strength [46]. They found that pure PMMA’s tensile strength rose from 5.45 MPa to 14.85 MPa at the maximum doping concentration.

Consequently, Figure 7D highlights the elongation at break, which provides insight into the ductility of the materials. Bare PMMA demonstrates a fracture strain of approximately 38.13%, whereas the composites with 25% and 50% Bi_2_O_3_-NPs exhibit a higher fracture strain, with values of 53.02% and 58.23%, respectively. These results suggest that the presence of Bi_2_O_3_-NPs improves the ductility of the composite materials, with the higher 50% nanoparticle concentration achieving the highest elongation at break, 1.53-fold higher than the bare PMMA. The addition of nanoparticles into polymer composites can influence ductility in various ways. The SEM and EDX characterization (Figure 4) illustrates the uniform dispersion of Bi_2_O_3_-NPs within the PMMA can enhance interfacial bonding via synergistic processes such as chemical interactions, mechanical interlocking, increased wettability, the manipulation of polymer chain arrangement, and charge transfer effects.

### 3.2. Radiation Shielding Properties of Bi_2_O_3_ Nanoparticle-Enhanced PMMA: Simulation and Experimental Results

#### 3.2.1. Evaluating Radiation Shielding Effectiveness Through XCOM Computational Model

Increasing the Bi_2_O_3_ content (25%, 50%, and 75%) enhances the coherent scattering cross-section, as seen by the upward shift in the curves as the Bi_2_O_3_ percentage increases. As increasing the Bi_2_O_3_ content from 25% to 75%, the coherent scattering cross-section rises proportionally, indicating that the scattering enhancement is directly linked to the proportion of Bi_2_O_3_ in the composite.

**Figure 8:**
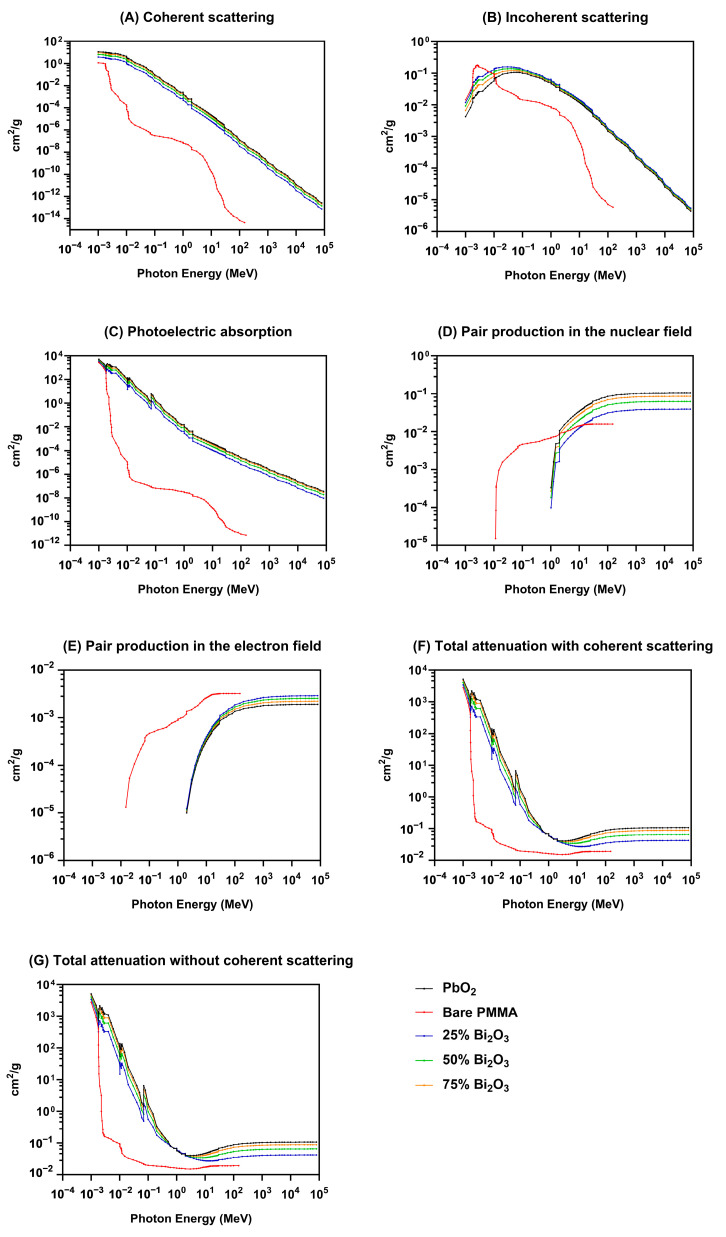
XCOM-based simulation of materials for radiation shielding. The graphs present the photon interaction coefficients for various materials across a range of photon energies: (**A**) coherent scattering, (**B**) incoherent scattering, (**C**) photoelectric absorption, (**D**) pair production in the nuclear field, (**E**) pair production in the electron field, (**F**) total attenuation with coherent scattering, (**G**) total attenuation without coherent scattering. Materials studied include lead oxide (PbO_2_), bare poly(methyl methacrylate) (PMMA), and PMMA composites containing 25%, 50%, and 75% bismuth oxide (Bi_2_O_3_).

The variation in incoherent scattering as a consequence of photon energy is shown in graph Figure 8B. PbO_2_ shows consistently high incoherent scattering values across the energy spectrum due to its high atomic number, increasing the probability of photon interactions. Bare PMMA’s incoherent scattering values are significantly lower than the other materials, particularly at higher photon energies. This is consistent with its low atomic number (Z), which reduces photon interactions. Adding Bi_2_O_3_ to PMMA progressively increases the incoherent scattering, with the magnitude directly proportional to the Bi_2_O_3_ concentration. This trend reflects the role of the bismuth high-Z element in enhancing photon interaction [47].

Figure 8C demonstrates PbO_2_ exhibits the highest photoelectric absorption values due to its high atomic number. The absorption decreases significantly with increasing photon energy, but its dominance is maintained over the other materials across the energy spectrum. The rapid decline in PMMA photoelectric absorption as photon energy increases is evident, following the characteristic inverse relationship between the photoelectric effect and photon energy (α Z^3^/E^3^). The addition of Bi_2_O_3_ to PMMA enhances the photoelectric absorption coefficients. Moreover, the higher concentrations of Bi_2_O_3_ (50% and 75%) exhibit a more significant absorption effect, approaching the values observed for PbO_2_.

The pair production in the nuclear field graph (Figure 8D) illustrates the variation in the pair production cross-section as a function of photon energy. The cross-section is represented in cm^2^/g, and the photon energy spans from 10^−2^ MeV to 10^5^ MeV on a logarithmic scale. All materials show a sharp decline in photoelectric absorption with increasing photon energy, consistent with the physics of photon–matter interactions, particularly for low-energy photons where the photoelectric effect is more significant. The PbO_2_ and Bi_2_O_3_-NPs composites exhibit higher pair production cross-sections due to their higher atomic numbers, particularly at photon energies exceeding 1 MeV.

Figure 8E illustrates the pair production in an electron field as a function of photon energy for various materials and composites. As can be seen, the pair production cross-section becomes significant at photon energies above 1 MeV, corresponding to the pair production energy threshold [48]. Below this threshold, the cross-section is negligible across all materials.

Additionally, the variation in total attenuation coefficients with coherent scattering for different materials as a function of photon energy is illustrated in Figure 8F. PbO_2_ exhibits the highest attenuation across the energy spectrum, owing to its higher density and atomic number compared to the PMMA composites. As shown, Bi_2_O_3_-NPs composites exhibit improved attenuation performance more so than bare PMMA, particularly at lower photon energies, highlighting the impact of Bi_2_O_3_ as a high-Z material in enhancing photon interaction probabilities. The attenuation trends align with the increasing Bi_2_O_3_ content, as demonstrated by the proportional enhancement in attenuation coefficients.

The total attenuation without coherent scattering (Figure 8G) presents the difference in total attenuation coefficients (cm^2^/g) as a function of photon energy. The logarithmic scales on both axes allow for the evaluation of attenuation trends over several orders of magnitude in energy and attenuation. The graph indicates Bi_2_O_3_-NPs composites that increase the attenuation performance, highlighting the role of Bi_2_O_3_ as a high-Z material, enhancing photon interaction probabilities, especially at low photon energies.

#### 3.2.2. I-131 Radiation Shielding Performance Analysis Using Phy-X/PSD Simulation

**Figure 9:**
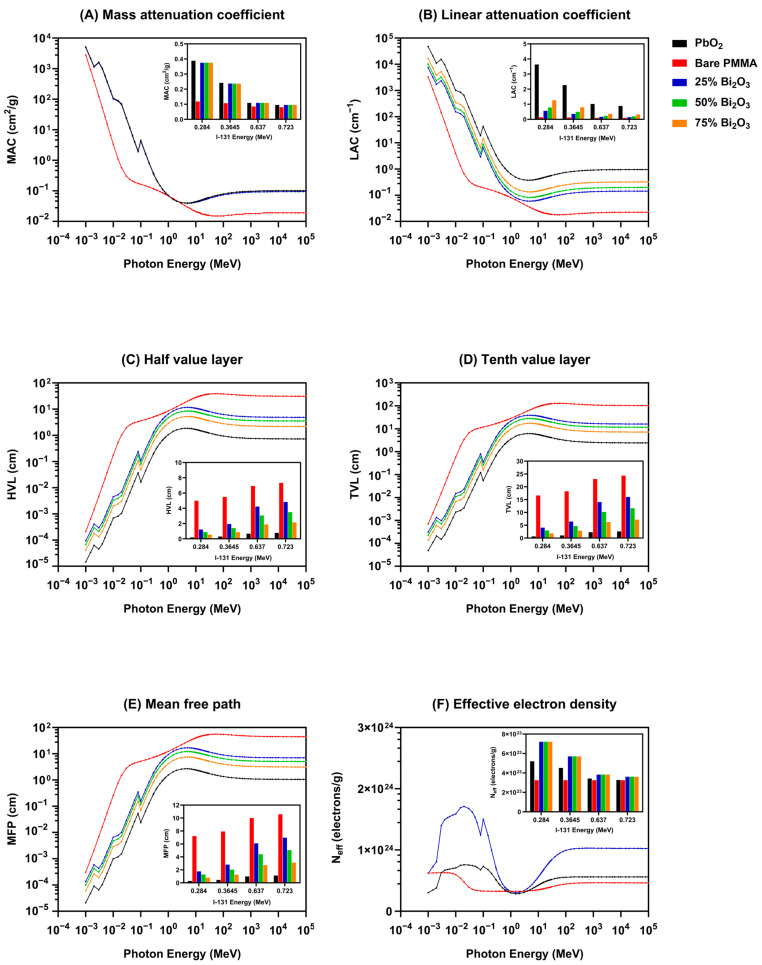

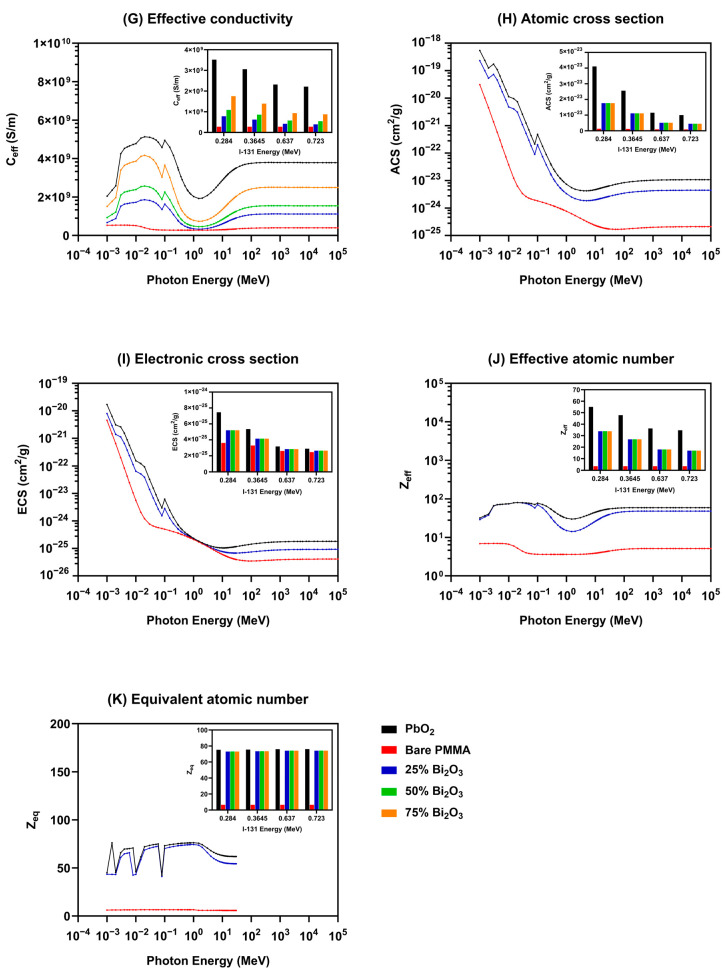
Phy-X/PSD-based simulation of materials for I-131 radiation shielding. The graphs illustrate key radiation shielding parameters for different materials across a range of photon energies: (**A**) mass attenuation coefficient (MAC), (**B**) linear attenuation coefficient (LAC), (**C**) half-value layer (HVL), (**D**) tenth-value layer (TVL), (**E**) mean free path (MFP), (**F**) effective electron density (N_eff_), (**G**) effective conductivity (C_eff_), (**H**) atomic cross-section (ACS), (**I**) electronic cross-section (ECS), (**J**) effective atomic number (Z_eff_), (**K**) equivalent atomic number (Z_eq_). Materials evaluated include lead oxide (PbO_2_), bare polymethyl methacrylate (PMMA), and PMMA composites with 25%, 50%, and 75% bismuth oxide (Bi_2_O_3_). The insets within the graphs provide comprehensive details on specific parameter values corresponding to photon energies pertinent to I-131 radiation shielding.

The shielding values determined with Phy-X/PSD software version 1.0 are presented in Figure 9. The graph (Figure 9A) illustrates the MAC as a function of photon energy and provides insights into the materials energy-dependent shielding efficiency of these materials. The MAC values become significantly higher at lower photon energy (below 0.1 MeV), signifying a pronounced photoelectric absorption impact [49]. The curves for all materials exhibit a sharp decline as photon energy increases, which aligns with the reduced dominance of photoelectric interactions.

The LAC values are presented across a wide photon energy range, spanning several orders of magnitude, with a logarithmic scale for both axes (Figure 9B). An inset provides a focused comparison of LAC values at specific photon energies corresponding to I-131 emission lines. PbO_2_ exhibits the highest LAC values across the entire energy spectrum, indicating its superior photon attenuation capability. However, incorporating Bi_2_O_3_ into PMMA significantly enhances its LAC, with the degree of enhancement proportional to the Bi_2_O_3_ concentration [50]. The 75% Bi_2_O_3_ composite shows the highest attenuation among the PMMA composite materials.

Figure 9C illustrates the relationship between the HVL and photon energy, and the inset provides detailed HVL values corresponding to photon energies associated with I-131 emissions. The HVL exhibits an upward trend with photon energy across all materials, signifying a decrease in attenuation efficiency at elevated photon energies due to the main effects of Compton scattering and pair creation. Notably, a significant increase in HVL is observed within the intermediate energy range, reflecting the transition from the photoelectric effect to Compton scattering. PbO_2_ exhibits the lowest HVL across all photon energies. Increasing the Bi_2_O_3_ concentration in PMMA significantly reduces the HVL. At 75% Bi_2_O_3_, the HVL approaches values closer to PbO_2_, especially at lower photon energies, indicating improved attenuation performance.

The TVL represents the material thickness that is required to reduce photon radiation intensity by 90% (Figure 9D). The analyzed materials include PbO_2_, bare PMMA, and PMMA composites with 25%, 50%, and 75% Bi_2_O_3_. As the Bi_2_O_3_ concentration increases (25% to 75%), the TVL decreases, confirming an enhanced attenuation capability with higher Bi_2_O_3_ content [51].

Figure 9E illustrates the MFP as a function of photon energy, the primary graph demonstrates the MFP over a wide energy range, and the inset provides a focused comparison at specific photon energies corresponding to Iodine-131. At lower photon energies, the MFP is significantly reduced for all materials, indicating high attenuation due to photoelectric absorption. As photon energy increases, MFP values rise steeply, reflecting the reduced dominance of photoelectric interactions and an increased contribution of Compton scattering. As can be seen, the PbO_2_- and Bi_2_O_3_-enhanced PMMA composites exhibit strong attenuation capabilities, particularly at low-to-moderate photon energies.

Figure 9F presents the N_eff_ as a function of photon energy, highlighting its energy-dependent variation. Neff exhibits significant variation for all materials across the photon energy range, particularly in the low-energy region. The effective electron density stabilizes at higher photon energies (>10^2^ MeV), indicating reduced dependency on the composition of materials at these energy levels.

Figure 9G illustrates the C_eff_ as a function of photon energy for bare PMMA, PbO_2_, and PMMA composites with varying percentages of Bi_2_O_3_ (25%, 50%, and 75%). The inset graph shows C_eff_ at I-131 energy levels (0.284, 0.3645, 0.637, and 0.723 MeV). PbO_2_ consistently exhibits the highest C_eff_ values, followed by PMMA composites with increasing Bi_2_O_3_ content (75%, 50%, and 25%). The findings indicate that the integration of Bi_2_O_3_, characterized by its high Z, into PMMA markedly enhances its effective conductivity. This improvement is attributed to the elevated atomic number and density of Bi_2_O_3_ (8.9 g/cm^3^), which promote increased photon interactions, predominantly through mechanisms such as Compton scattering and photoelectric absorption.

Figure 9H depicts the ACS as a function of photon energy, indicating the ACS values that show a distinct dependency on photon energy across the range 10^−4^ MeV to 10^6^ MeV. The addition of Bi_2_O_3_ to PMMA significantly improves ACS across all energy ranges. This enhancement is more pronounced at lower energies, where photoelectric absorption dominates.

The ECS as a function of photon energy is illustrated in Figure 9I. The ECS decreases with increasing photon energy, showing distinct regions corresponding to photoelectric absorption, Compton scattering, and pair production processes. As the weight percentage of Bi_2_O_3_ increases (from 25% to 75%), the ECS values rise progressively, particularly at lower photon energies. This is attributable to the incorporation of bismuth, a high-Z element, enhancing photoelectric absorption and overall attenuation.

Figure 9J illustrates the variation of the Z_eff_ as a function of photon energy. At low photon energies (below 0.1 MeV), the Z_eff_ for PbO_2_ and Bi_2_O_3_-PMMA composites demonstrates significantly higher values compared to bare PMMA. This is indicative of enhanced photoelectric absorption due to the high atomic numbers of Pb and Bi. In the intermediate energy range (0.1 to 10 MeV), the Z_eff_ values for all materials converge, indicating the dominance of Compton scattering, which is less dependent on atomic number. At high energies (beyond 10^2^ MeV), Z_eff_ slightly increases for materials containing high-Z elements, reflecting pair production phenomena. The Z_eff_ for 75% Bi_2_O_3_-PMMA is constantly closer to PbO_2_, demonstrating the efficacy of high bismuth content in enhancing attenuation properties.

Figure 9K illustrates the Z_eq_ and represents the equivalent atomic number as a function of photon energy, spanning a wide range of energies (10^−4^ MeV to 10^2^ MeV). The inset highlights the Z_eq_ values at specific photon energies corresponding to I-131 emission energies (0.284, 0.3645, 0.637, and 0.723 MeV). The Z_eq_ varies significantly across the photon energy range. At lower photon energies (<10^−2^ MeV), Z_eq_ is higher, particularly for PbO_2_, indicating strong photoelectric absorption dominance. As photon energy increases (>10 MeV), the Z_eq_ decreases, which can be attributed to the predominance of Compton scattering over photoelectric absorption. The Z_eq_ increases with the percentage of Bi_2_O_3_ in PMMA. This is particularly noticeable at low photon energies, where the photoelectric effect is sensitive to the atomic number. The 75% Bi_2_O_3_ composite approaches the performance of PbO_2_. In the range of ~0.01 to 10 MeV, a distinct dip in Z_eq_ is observed for all materials. This region corresponds to the energy range where the interaction processes transition from photoelectric dominance to Compton scattering dominance. For PbO_2_ and Bi_2_O_3_ composites, this dip is less pronounced compared to bare PMMA, indicating higher Z_eq_ retention due to their heavier elements.

#### 3.2.3. Advanced Composite Materials for Effective I-131 Radiation Shielding

**Figure 10:**
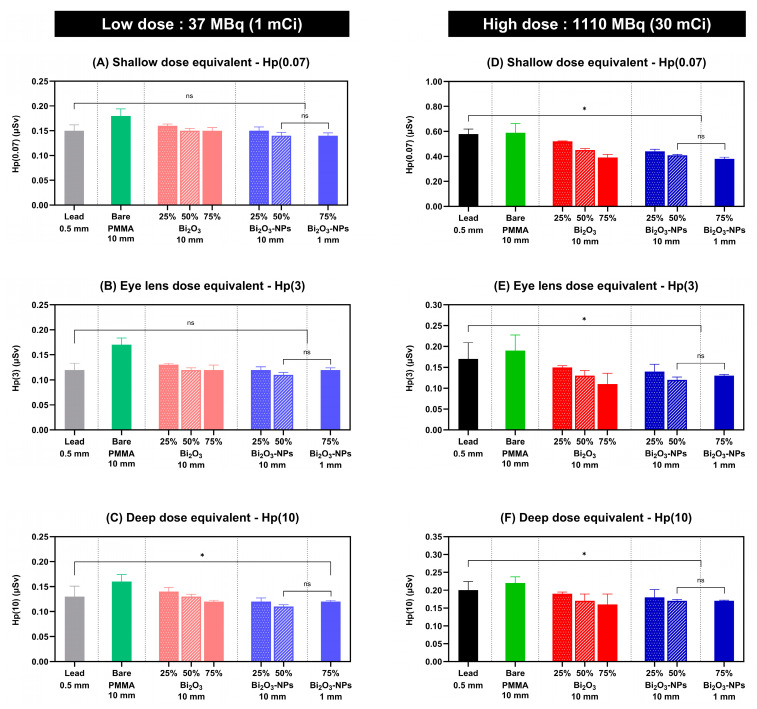
Radiation dose equivalents at low and high doses for various shielding materials and compositions. (**A**) Shallow-dose equivalent (Hp(0.07)), (**B**) eye lens dose equivalent (Hp(3)), and (**C**) deep dose equivalent (Hp(10)) were evaluated at a low dose (37 MBq). (**D**) Shallow-dose equivalent (Hp(0.07)), (**E**) eye lens dose equivalent (Hp(3)), and (**F**) deep-dose equivalent (Hp(10)) were analyzed at a high dose (1110 MBq). The shielding materials tested included lead (0.5 mm), bare PMMA (10 mm), and Bi_2_O_3_ and Bi_2_O_3_ NPs at varying concentrations (25%, 50%, and 75%) with thicknesses of 10 mm and 1 mm. Data represent mean ± standard deviation (*n* = 3). Statistical significance is indicated by * *p* < 0.05, while “ns” denotes no significant difference.

The skin shallow-dose equivalent, Hp(0.07), used for various shielding configurations under a low radiation dose of 37 MBq (1 mCi), is shown in Figure 10A. PbO_2_ is known for its photoelectric absorption and high atomic number and density-increasing photon interaction, causing well-documented attenuation [52]. PbO_2_ (0.5 mm) provided a baseline for shielding with a mean dose of 0.15 μSv. PMMA 50% Bi_2_O_3_ (10 mm thickness) resulted in a slightly lower mean dose of 0.14 μSv. In comparison, the PMMA with the addition of 75% Bi_2_O_3_-NPs with only a 1 mm thickness resulted in a mean dose of 0.14 µSv that was lower than PbO_2_.

Eye lens dose equivalent Hp(3) results are illustrated in Figure 10B, with PbO_2_ (0.5 mm) resulting in a mean dose of 0.13 µSv. The PMMA with 25% Bi_2_O_3_ (10 mm thickness) has a mean dose of 0.12 µSv, and PMMA with 50% and 75% Bi_2_O_3_ (10 mm thickness) has a mean dose of 0.12 µSv. The PMMA with the addition of 25% Bi_2_O_3_-NPs (with a 10 mm thickness) resulted in a mean dose of 0.12 µSv, the PMMA 50% Bi_2_O_3_-NPs resulted in a mean dose of 0.11 µSv, and PMMA 75% Bi_2_O_3_-NPs with a 1 mm thickness resulted in a mean dose of 0.12 µSv. The PMMA Bi_2_O_3_ NP-enhanced performance is likely due to the nanoparticle’s higher surface area and improved the homogeneity of nanoparticles, which enhances photon attenuation efficiency.

Figure 10C represents the deep-dose equivalent of the whole body (Hp(10)) used for various materials and configurations at a low radiation dose of 37 MBq (1 mCi). PbO_2_ (0.5 mm) demonstrates significantly reduced Hp(10) with a mean dose of 0.13 µSv compared to bare a PMMA mean dose of 0.16 µSv, affirming its effectiveness as a shielding material. Augmenting the Bi_2_O_3_ concentrations in PMMA composites from 25% to 75% within a 10 mm layer leads to a progressive decline in Hp(10), 0.14 µSv at 25%, 0.13 µSv at 50%, and 0.12 µSv at 75%. The results signify a positive relationship between Bi_2_O_3_ concentration and shielding efficacy. Furthermore, the PMMA (10 mm thickness) enhanced with 25% Bi_2_O_3_-NPs resulted in a mean dose of 0.12 µSv that was lower than PbO_2_. Adding 25% Bi_2_O_3_-NPs to the PMMA (10 mm thickness) resulted in a mean dose of 0.12 µSv, which is lower than the PbO_2_ mean dose of 0.13 µSv. Similarly, the PMMA with only 1 mm thickness with 75% Bi_2_O_3_-NPs resulted in a mean dose of 0.12 µSv.

The shallow-dose equivalent Hp(0.07) for the different shielding materials exposed to a high dose of 1110 MBq (30 mCi) of radiation is presented in Figure 10D. PbO_2_ has a mean dose of 0.58 µSv, and the PMMA Bi_2_O_3_ composites resulted in mean doses lower than the PbO_2_. Enhancing the PMMA (10 mm thickness) with the addition of 25% Bi_2_O_3_-NPs reduced the mean dose to 0.44 µSv, 50% Bi_2_O_3_-NPs reduced the mean dose to 0.41 µSv, and the PMMA (1 mm thickness) enhanced with 75% Bi_2_O_3_-NPs, achieving a mean dose of 0.38 µSv.

The comparison of eye lens dose equivalent, denoted as Hp(3), is presented in Figure 10E. The mean PbO_2_ (0.5 mm) dose was 0.17 µSv, and bare PMMA (10 mm) was 0.19 µSv. The addition of Bi_2_O_3_ in PMMA at 25%, 50%, and 75% concentrations shows a clear dose reduction trend, with higher concentrations providing enhanced protection. Incorporating 25% Bi_2_O_3_-NPs into 10 mm thick PMMA decreased the mean dosage to 0.14 µSv, while 50% Bi_2_O_3_-NPs lowered it to 0.12 µSv. Additionally, 1 mm thick PMMA augmented with 75% Bi_2_O_3_-NPs resulted in a mean dose of 0.13 µSv.

Figure 10F illustrates the comparison of a high-dose whole-body equivalent Hp(10). PbO_2_ (0.5 mm) shows a low Hp(10) value with a mean dose of 0.20 μSv, displaying its well-known efficacy as a radiation shield due to its high atomic number (Z = 82) and density. However, the PMMA (10 mm) Bi_2_O_3_ composites mean dose rates are lower, 25% Bi_2_O_3_ has a mean dose of 0.19 µSv, 50% Bi_2_O_3_ has a mean dose of 0.17 µSv, and 75% Bi_2_O_3_ has a mean dose of 0.16 µSv. Furthermore, the PMMA (10 mm) enhanced with Bi_2_O_3_-NPs resulted in lower mean dose rates, with 25% resulting in a mean dose of 18 µSv and 50% Bi_2_O_3_-NPs resulting in a mean dose rate of 17 µSv. Notably, the PMMA with a 1 mm thickness enhanced with 75% Bi_2_O_3_-NPs achieved a mean dose of 17 µSv (with *p* < 0.05 marking a significant difference to PbO_2_).

In summary, the PMMA (1 mm thickness) enhanced with 75% Bi_2_O_3_-NPs at a low radiation dose of 37 MBq (1 mCi) achieved lower mean doses than PbO_2_. Additionally, at a higher dose of 1110 MBq (30 mCi), the PMMA enhanced with 75% Bi_2_O_3_-NPs resulted in a significantly lower mean dose than PbO_2_.

## 4. Discussion

The characterization of Bi_2_O_3_ NP-enhanced PMMA determined that Bi_2_O_3_-NPs have a considerably smaller z-average diameter of 490 nm than Bi_2_O_3_ 1018 nm, resulting in an enhanced surface area (Figure 3). This increase in the surface area of the Bi_2_O_3_-NPs facilitates the improved interaction and dispersion of the nanoparticles within the PMMA matrix, which is critical for achieving a homogeneous composite structure. The combination of a smaller nanoparticle size, narrower PDI, and higher negative zeta potential contributes to the enhanced mechanical performance of the PMMA/Bi_2_O_3_ composite. A number of studies have shown that the consistent distribution of high-density components in polymer composites increases gamma radiation attenuation, resulting in improved shielding efficacy [53,54,55]. The results of the quantitative EDX elemental analysis (Figure 4) illustrate the addition of Bi_2_O_3_-NPs to enhance gamma radiation shielding properties, given bismuth’s high atomic number and strong attenuation capabilities [41]. Jayakumar et al. reported that nanocomposites based on Bi_2_O_3_ combined with silicone polymers with increased density demonstrated superior attenuation to other nanocomposites [56]. Furthermore, XRD is increasingly utilized to characterize structures illustrating the integration of the Bi_2_O_3_-NPs into the PMMA matrix, preserving the polymer’s amorphous characteristics while incorporating the crystalline nanoparticles (Figure 5) [57]. The XRD pattern for bulk Bi_2_O_3_ exhibits relatively broad diffraction peaks, suggesting a lower degree of crystallinity compared to the Bi_2_O_3_ nanoparticles. The diffraction peaks are characteristic of the monoclinic phase (α-Bi_2_O_3_), with diffraction peaks at around 20–25° and 30–35° 2θ. The broader, less intense peaks of Bi_2_O_3_ indicate larger particle sizes and less defined crystal structures than the Bi_2_O_3_ nanoparticles. The Bi_2_O_3_-NPs XRD pattern corresponds to the monoclinic phase (α-Bi_2_O_3_), with sharper, more intense peaks, suggesting a higher degree of crystallinity, indicating that the Bi_2_O_3_-NPs are well ordered in terms of their crystalline structure [58]. The highly ordered arrangement in crystalline Bi_2_O_3_ minimizes defects and voids, which are common in amorphous materials. These defects can reduce the effective shielding capability by introducing pathways of lower resistance for radiation penetration. High crystallinity ensures consistent performance under prolonged radiation exposure [59]. Additionally, crystalline Bi_2_O_3_ has a higher density due to its tighter atomic structure; this increased density correlates directly with improved gamma-ray attenuation, as denser materials offer more nuclei per unit volume for interaction. El-Bashir et al. studied the addition of Bi_2_O_3_ in phosphate glass and established densities increased due to Bi_2_O_3_ heavier Bi atoms [60].

Notably, the addition of Bi_2_O_3_-NPs to the PMMA result in a composite material with a balance of structural flexibility, enhanced functionality, and tunability. In particular, the high atomic number of bismuth in Bi_2_O_3_-NPs contributes to the material’s ability to attenuate gamma radiation, making the Bi_2_O_3_ NP-enhanced PMMA composite a promising candidate for I-131 radiation shielding applications [61]. Furthermore, the FTIR-based chemical bonding analysis of Bi_2_O_3_-NPs-enhanced PMMA composites (Figure 6) confirms the successful formation of Bi_2_O_3_-NPs, with characteristic Bi–O–Bi and Bi–O bond stretching vibrations indicating the oxide framework. The FTIR analysis of the dispersion quality of Bi_2_O_3_ nanoparticles in PMMA suggests the characteristic peaks with small changes, indicating that nanoparticles are physically merged rather than phase-separated. Minor shifts in C=O and C–O–C stretching suggest physical interactions between Bi_2_O_3_-NPs and PMMA. Moreover, the lack of peak widening or additional peaks suggests that Bi_2_O_3_-NPs are evenly distributed and not agglomerated.

In addition, the tensile properties of Bi_2_O_3_ Figure 7B,C reveal that incorporating Bi_2_O_3_ may affect the composite’s ability to withstand applied forces, possibly due to high nanoparticle concentration in the polymer matrix, causing stress concentrators and reducing overall strength [62,63]. Due to van der Waals forces and poor dispersion, nanoparticles cluster at high concentrations. When mechanically loaded, these agglomerates concentrate tension in the matrix. The poorly dispersed nanoparticles reduce stress transfer between the polymer and nanoparticles, weakening the composite. Additionally, high nanoparticle loading may exceed the polymer’s wetting capabilities, posing a risk to interfacial bonding [64,65]. Cracks can start at weak surfaces under stress, lowering material mechanical integrity [66]. A high nanoparticle content stiffens the matrix, minimizing plastic deformation, and a less ductile matrix releases less energy during fracture, making composites more likely to fail [62]. Nonetheless, a study by Mahmood et al. successfully enhanced the mechanical properties of PMMA, reinforcing it with a Bi_2_O_3_:Fe_2_O_3_ nanoparticle composite that significantly improved its mechanical properties and radiation shielding capabilities [46]. Furthermore, a study by Yasser et al. determined that the addition of Bi_2_O_3_ nanoparticles enhanced the compressive strength of PMMA composites [67].

The ductility mechanical test determined the elongation at break of the Bi_2_O_3_-NPs, indicating that the fracture strain (Figure 7D) was significantly higher than bare PMMA. This suggests the integration of Bi_2_O_3_-NPs into PMMA enhanced interaction and dispersion, improving interfacial bonding and enhancing the PMMA characteristics. Bi_2_O_3_-NPs have high surface energy owing to their compact size and high surface-area-to-volume ratio, increasing chemical interactions between nanoparticle surfaces and PMMA matrix. Hydroxyl (–OH) groups on Bi_2_O_3_ surfaces may create hydrogen bonds or van der Waals interactions with PMMA ester groups (–COOCH_3_). Bi_2_O_3_ nanoparticle size and shape are critical for mechanical interlocking in polymer matrices. Uniformly scattered nanoparticles may function as reinforcement fillers to improve frictional resistance and limit polymer chain mobility at the interface. This effect improves interface load transfer efficiency, tensile strength, impact resistance, and toughness. The addition of Bi_2_O_3_-NPs changes the surface energy of PMMA, increasing wettability and stickiness, increasing polymer chain–nanoparticle bonding, eliminating interfacial gaps and mechanical stress debonding. Additionally, the high dielectric constant of Bi_2_O_3_-NPs influences polarization interactions in the PMMA matrix. Increasing the electrostatic interactions between polymer chains and nanoparticles and strengthening interfacial adhesion.

The mechanical properties findings suggest that while the addition of Bi_2_O_3_-NPs decreases the overall tensile strength of PMMA, it enhances its flexibility, making it more resistant to deformation before failure. This trade-off between strength and ductility could be advantageous in applications requiring impact resistance or structural resilience under mechanical stress.

In addition, the radiation shielding properties of Bi_2_O_3_ NP-enhanced PMMA (Figure 8) XCOM computational model illustrate the reduction in coherent scattering cross-section with increasing photon energy [68]. Coherent scattering dominates at lower photon energies (keV range) due to the stronger interaction of low-energy photons with atomic electrons. As photon energy increases, the photon wavelength decreases and reduces the probability of interaction, resulting in a monotonic decline in the scattering cross-section, as seen in all curves (Figure 8A). Similarly, the addition of Bi_2_O_3_ to PMMA significantly enhances photon interaction properties across all interaction mechanisms. It is directly proportional to the Bi_2_O_3_ concentration in the composites, with higher concentrations approaching the performance of PbO_2_. The XCOM computational model results underscore the effectiveness of Bi_2_O_3_ as a high-Z additive for improving the radiation attenuation capabilities of PMMA-based materials, making them viable for applications requiring enhanced photon shielding or interaction.

The Phy-X/PSD simulation findings underscore the critical role of high-Z materials in radiation shielding. While PbO_2_ remains superior, PMMA composites with Bi_2_O_3_ offer a tunable alternative with significantly improved performance proportional to Bi_2_O_3_ content. The results shown in Figure 9 demonstrate that the 75% Bi_2_O_3_ composite constantly approaches values closer to PbO_2_. The Phy-X/PSD analysis draws attention to the superior shielding efficacy of PbO_2_ across all photon energies, making it an effective material. However, incorporating Bi_2_O_3_ into PMMA significantly enhances its attenuation performance, with higher concentrations yielding more significant radiation shielding. These findings demonstrate the potential of Bi_2_O_3_-PMMA composites in radiation shielding, particularly at higher concentrations, aligning closely with the performance of PbO_2_.

The PMMA Bi_2_O_3_-NPs composites exhibited notably superior radiation shielding performance compared to PbO_2_ in both low (37 MBq) and high (1110 MBq) radiation exposure scenarios. Specifically, for the shallow-dose equivalent Hp(0.07), the PMMA composite containing 75% Bi_2_O_3_-NPs (1 mm thickness) recorded significantly lower mean doses, measuring just 0.14 µSv and 0.38 µSv at 37 MBq and 1110 MBq, respectively, outpacing PbO_2_. Additionally, the eye lens dose equivalent Hp(3) demonstrated a considerable reduction with increased Bi_2_O_3_ concentration; the 50% Bi_2_O_3_ NP composite (10 mm thickness) achieved an impressively low dose of 0.12 µSv at both 37 MBq and 1110 MBq, exceeding the attenuation capabilities of PbO_2_. The lower standard deviation values for the PMMA Bi_2_O_3_-NPs composites further highlight their reliability and consistent shielding efficacy. Significantly, the enhanced dose attenuation accomplished with reduced material thickness and weight positions these composites as promising candidates for advanced radiation shielding applications, meeting critical needs for lightweight and efficient radiation protection materials.

Moreover, there is increasing concern regarding whether long-term low-dose radiation exposure increases carcinogenesis and the risk of genetic instability, particularly to medical professionals [69]. Non-cancer health effects include cardiovascular disease (predominantly ischemic heart disease) [70], cataracts [71], immune function disorders [72], and neurological illnesses [73]. Research has demonstrated that exposure to gamma radiation at doses comparable to those used in cancer treatment induces DNA fragmentation. This fragmentation leads to a reduction in the quantity of detectable genetic loci [74]. Additionally, Ma et al. compared chronic low-dose γ-irradiation at low-dose-rate to generate cognitive impairment to impairment from high-dose-rate exposure. They found, in comparison to the high-dose-rate, that the low-dose-rate resulted in more pronounced cognitive deficits, potentially implicating the PI3K/Akt signaling pathway [75]. Despite the use of medical lead shielding, the significant penetrative capacity of gamma rays renders total protection for medical personnel in challenging clinical environments. As a result, this creates a continuous low-dose gamma radiation exposure environment [76]. Notably, the findings of an epidemiological study suggest that Parkinson’s disease correlates with sustained exposure to γ-rays [77]. Due to the concern regarding low-dose radiation in 2010, a European radiation protection research platform called MELODI was founded [78]. To study ionizing radiation health issues from low-dose radiation, we recommend conducting research to identify the relationship between cancer risk on dose and dose rate, the non-cancer consequences of radiation, and individual sensitivity to radiation. Although the robust radiation protection measures are considered as being satisfactory, there remains scientific uncertainty about the health hazards from low doses and/or low-dose rates [79].

Although lead aprons are the standard for radiation protection, their weight may induce pain and other health complications, resulting in inconsistent usage by medical personnel [80,81]. The advancement of lighter protective materials aims to address these issues, enhancing compliance and maintaining safety in radiological settings. Consequently, in comparison to lead, polymeric composites and nanocomposites serve as an effective, lightweight shield against gamma radiation.

## 5. Conclusions

The integration of Bi_2_O_3_-NPs into PMMA composites demonstrates a significant enhancement in the material’s gamma radiation shielding properties, structural integrity, and functional versatility. The reduced z-average diameter of Bi_2_O_3_-NPs, compared to bulk Bi_2_O_3_, results in an increased surface area, promoting better dispersion within the PMMA matrix and ensuring a homogeneous composite structure. This uniformity is critical for optimizing the interaction between high-density components and the polymer matrix, as supported by the enhanced gamma radiation attenuation observed in multiple studies. Quantitative analyses, including EDX, XRD, and computational modeling, confirm the superior attenuation capabilities of Bi_2_O_3_-enhanced PMMA composites, attributed to the high atomic number, density, and crystallinity of the Bi_2_O_3_-NPs. The XRD patterns highlight the ordered crystalline structure of Bi_2_O_3_-NPs, which minimizes structural defects and enhances gamma-ray attenuation by providing more nuclei for photon interaction. FTIR analysis further substantiates the chemical compatibility and effective integration of the nanoparticles within the PMMA matrix. The computational simulations (XCOM and Phy-X/PSD) highlight the effectiveness of Bi_2_O_3_ as a high-Z additive, with higher nanoparticle concentrations significantly enhancing photon shielding capabilities. While PbO_2_ remains the most effective shielding material, the tunability and improved performance of PMMA-Bi_2_O_3_-NPs composites offer a promising lightweight alternative for applications requiring effective radiation attenuation. Despite the improvements in shielding properties, the mechanical limitations at higher nanoparticle concentrations, including reduced stress transfer and the potential formation of stress concentrators, highlight the need for nanoparticle dispersion and loading optimization. Addressing these challenges could further enhance the mechanical integrity and durability of the composites under applied forces.

In the context of gamma radiation’s detrimental health impacts, including carcinogenesis and cardiovascular, neurological, and immune system disorders, the development of lightweight, efficient shielding materials such as PMMA-Bi_2_O_3_-NPs composites represent a critical advancement. These materials offer a practical alternative to lead-based shielding, reducing exposure risks while maintaining functionality and ease of application. Future research should continue to refine these composites to optimize their mechanical and shielding properties, ensuring their viability in diverse clinical and industrial applications.

## Data Availability

The original contributions presented in this study are included in the article material. Further inquiries can be directed to the corresponding author.
